# 

**Published:** 1899-01

**Authors:** 


### INDEX

TO THE

### HOMŒOPATHIC PHYSICIAN.

### VOLUME XIX, 1899.

PAGE

Ac[illegible]e Powder Measure ... 194
Acromegaly and Vaccination ... 365
Adams, Edward. Progress of Our School. Has it been what it should have been? ... 199
Address of the President of the International Hahnemannian Association ... 299
Air, Liquefied ... 417
Alcohol and Insanity ... 463
Allen, Dr. J. Henry. Removal ... 247
Amalgam Fillings in Teeth ... 260
American Institute of Homœopathy, 37, 196, 198, 425, 506
American Institute of Homœopathy Goes to Paris in 1900 ... 506
American People: Address to ... 465
Anæmia, Pernicious ... 427
Anæsthetic, A New ... 96
Antikamnia Chemical Co ... 246
Antitoxin ... 43, 249
Antitoxin Treatment, Relation of to Homœopathy. Review ... 518
Are Readers' Eyes Worth Saving? ... 189

Bacteria, Diagnostic Value of ... 149
Bacteriology as a Fad ... 474, 488
Bailey, B. F. American Institute of Homœopathy ... 39, 196, 343, 425
Bee-line Therapia and Repertory. Review ... 516
Benevolence and Philanthropy, Chance for ... 461
Beaumont, Dr. E. S. Removal ... 435
Birds, Treatment of Cage. Review ... 434
Bladder, Accidental Wounds of Female ... 511
Bœricke, Runyon & Ernesty ... 42
Book Notices ... 39, 93, 193, 246, 297, 341, 390, 430, 473, 515, 563
Broken Doses ... 298
Bubonic Plague ... 436, 518
Bunsen, Professor ... 514

Calcarea Case ... 191
Calendula Dressing ... 387
Carleton, E. Epilepsy ... 109

PAGE

Castor-oil Beans ... 41
Castration of Idiots ... 339
Cat, the Domestic ... 435
Catarrh, Chronic Naso-pharyngeal, 475, 519, 520
Change of Life in Women. Review ... 473
Chemists Order Snakes ... 345
Children's Paradise ... 363
Christian Life, The. Review ... 433
Chronic Catarrh Cured ... 475
Clinical Cases ... 145, 215, 217
Close, Stuart. Clinical Cases ... 217
What is the Vital Force ... 48
Consumption and Vaccination ... 390
Corsets in Russia ... 194
Custis, Dr. J. B. Gregg. Removal ... 343
Cyclopedia of Drug Pathogenesy. Review ... 564

Davis, F. S. Clinical Cases ... 215
Nasal Polypus ... 116
Susceptibility to Disease ... 7
Death Came While Trying to Save ... 245
Diet ... 362
Diphtheria, Bacteriological Diagnosis of ... 96
Diphtheria with Cases ... 156
Diphtheria, Homœopathic Therapeutics of. Review ... 93
Directory, British Colonial and Continental. Review ... 94
Dog Book. Review ... 391
Dog, The Toy ... 392
Drug Action ... 527
Drug Fxperience. A. G. Downer ... 522
Drug Intemperance ... 245

Eczema, Whortleberry in ... 467
Editorial ... 1, 45, 143, 195, 249, 347, 477, 521
Electrical Saws ... 346
Enzym ... 547
Epidemics, Endemics and Contagions, 479
Epilepsy ... 109, 383
Examining Board, Homœopathic Medical, of Pennsylvania ... 247

PAGE
Farley, Dr. Walter. In Memoriam... 336
Field *vs.* McGlumphy... 330
Fincke, B. Commentaries on Hahnemann's *Organon*... 348
International Hahnemann Association... 191
Foreign Bodies in Nasal Fossæ, New Method of Extracting... 472
Foster, Dr. Wm. D... 435
Fowler, Dr. Samuel Mills. In Memoriam... 332
Fun for Doctors...44, 346, 476

Garrison, Dr. John B. American Institute of Homœopathy... 508
Genito-Urinary Tract, Rational Treatment of Acute and Chronic Diseases of... 193
Genito-Urinary Tract, Surgical diseases of. Review... 564
Gilbert, Charles B. Nastiness... 546
Gladstone's Long Life, Secret of... 278
Glonoine, Proving of... 464
Glycerin, Death from Intrauterine Injection of... 423
Gonorrhœa, Hot Water in Treatment of... 97
Good Thing Mother was at Home... 436
Gospel of Hahnemann. How are we Proclaiming it?... 83
Gynæcology and Therapeutics. Conservative. Review... 341

Hahnemann Medical College of Philadelphia: Alumni Association... 141
Hahnemann Monument... 36, 138, 509
Hahnemann's *Organon*, Commentaries on... 348
Hahnemann's Tomb...296, 559
Hay Fever... 344
Hering's Repertory of Tooth-ache..521, 529
Hiller, D. A. Are Readers' Eyes Worth Saving?... 189
Hollingsworth, S. G. Drug Action... 527
Homœopathic Medical College of Missouri... 295
Homœopathic Medical Society of New York...382, 517
Homœopathic Therapeutics, Essentials of. Review... 39
Howard, C. C. Clinical Cases... 153
Hoyne, Temple S. In Memoriam... 131
Huffman, J. E. A Calcarea Case... 191
Miscarriage Prevented... 133
Painful Menstruation... 134

Ingersol, Col., Death of... 388
Inoculation and Vivisection... 144
Intermittent Fever...134, 252
International Congress...47, 545
International Hahnemannian Association...45, 191, 195, 347, 359, 379
International Hahnemannian Association, Address of President... 299
*International Medical Annual,* for 1899, Review...95, 246
Isolation of School Children: Period of... 386

James, B. W. Hahnemann Monument. 36
James, Bushrod W. Hahnemann's Tomb... 296

PAGE
James, Walter M. Address to International Hahnemannian Association... 299
Hering's Repertory of Tooth-ache, 521
Inoculation and Vivisection... 144
International Hahnemannian Association...45, 195, 347
Kali-bichrom... 477
Notice to Subscribers... 143
Puls... 1
Use of Antitoxin... 249

Kali-Bichrom... 477
Kennedy, A. L. The Gospel of Hahnemann, How are We Proclaiming It?... 83
Rheumatism... 99
Keynotes and Characteristics. H. C. Allen. Review... 40, 565
Kimball, S. A. Diphtheria with Cases. 156
Kimmel, Dr. Removal... 344

Laughter Cure... 389
Leaders in Homœopathic Therapeutics. Review of... 431
Left-handedness: Frequency of... 476
Leprosy... 436
Leverson, M. R. Acromegaly and Vaccination... 365
Biographical Memoir of Wm. Tebb...311, 407
Introductory Memoir of Mr. Wm. Tebb... 279
Vaccination in the Light of the Royal British Commission... 17, 120, 167, 224, 454, 489, 539
A Warning... 337
Lockjaw from Vaccination... 458
Logic of Figures. Review... 517
Ludlam, Dr. Reuben. Death of...245, 422

Marchand's Preparations... 97
Materia Medica and Therapeutics of Rare Homœopathic Remedies. Review... 518
Mather, Dr. E. Removal... 474
Medical Reciprocity... 558
Menninger, C. F. Raison d'être... 272
Menstruation: Painful... 134
Mercurials Differentiated... 265
Mineral Products of the United States. Review... 432
Miscarriage Prevented... 133
Morgan, W. L. Amalgam Fillings... 260
Clinical Cases... 145
Diagnostic Value of Bacteria... 149
Epidemics, Endemics, and Contagions... 479
Some Morbific Agency Inimical to Life: What Is It?... 77
Morphia Habit: Rational Treatment of... 505
Morphine Poisoning. Potassium-permang. in... 44
Mortality from Wounds... 42
Morrell, Charles B. Calendula Dressing... 387
Mud Baths... 384
Munson & Co. Removal of... 298

Nasal Polypus... 116
Nastiness... 546
Needle: Simple Method of Removing, 344
Newer Remedies. Review... 297

PAGE
New York Homœopathic Medical College.....41, 142
Notice to Subscribers..... 143
Notes and Notices...41, 96, 193, 297, 343, 392, 435, 474, 519

Obituary..... 141
Obstetrics and Obstetrical Surgery, 393, 437
Obstetrics: Text-book of. Review..... 563
O'Connor, Dr. Joseph. Removal..... 392
Opening for Young Physician..... 381
Orificial Surgery..... 380
Ovaries: Excision of..... 135
Ovariotomy Averted. Review..... 193

Patch, F. W. Case of Threatened Ththisis Pulmonalis..... 212
Intermittent Fever..... 252
Pease, F. O. Mercurials Differentiated..... 265
Peter, L. C. Rumination in a Boy..... 419
Philippines; Story of..... 141
Phonendoscope and Its Application. Review..... 94
Phthisis Pulmonalis. A Case of Threatened..... 212
Physician's Visiting List for 1900, Review..... 519
Picric Acid in Treatment of Burns..... 297
Pins: A Consumer of..... 424
Porcelain Painter's Son. Review..... 93
Poultry Book..... 391
Poultry Doctor. Review..... 434
Progress of Our School..... 199
Psychology and Mental Disease, Primer of. Review..... 40
Pulsatilla. Walter M. James..... 1
Pyrosis Chronic..... 248

Raison d'être..... 272
Remedies: New, Old and Forgotten. Review..... 566
Renal Therapeutics. Review..... 516
Repertory of Back...22, 174, 229, 288, 317, 366, 468, 497, 549
Rheumatism..... 99
Rumination in a Boy of Nine Years..... 419
Sanitarium; Dr. Givens'..... 141
Schussler: Twelve Tissue Remedies of. Review..... 430
Scientific American..... 142
Serum Therapy..... 98
Sewage in India; Disposal of..... 510
Sexual Instinct. Review..... 390

PAGE
Sexual Disordess of Men. Practical Treatise on.....82, 566
Skin-grafting Superseded by Skin Propagation..... 56
Smith, Emmett L. Bacteriology as a Fad..... 488
Society of Homœopathicians. 2, 48, 99, 145, 199, 252
Some Morbific Agency Inimical to Life. What is it?..... 77
Storer, Dr. John. Removal..... 247
Surgeon, First a, and then a Son, Clinically..... 422
Susceptibility to Disease. F. S. Davis.. 7

Talbot, I. T. In Memoriam.....381, 418
Tebb, Mr. William. Biographical Memoir.....279, 311, 407
Testifying, Exemption from..... 436
Three Thousand Questions on Medical Subjects. Review..... 95
Tight Lacing..... 91
Tooth-ache.....521, 529
Tooth-ache Notes..... 538
Tooting..... 97
Traumatic Ulceration..... 561
Triumphs of Modern Surgery..... 345
Tuberculosis..... 137

Underwood, M. F. Children's Paradise..... 363
Urinary Organs and Prostate Gland, Repertory of. Review..... 515

Vaccination..... 136
Vaccination in the Light of the Royal British Commission. 17, 121, 167, 224, 454, 489, 539
Vondergoltz, Eric. Obstetrics and Obstetrical Surgery.....393, 437

Waring, Colonel,..... 287
Warning, A..... 337
What is the Vital Force?..... 48
Whitman, J. A. Diet..... 362
Intermittent Fever..... 134
Removal of..... 193
Williams, Mary I. H. Excision of Ovaries..... 135
Wilsey, E. H. Repertory of the Back. 22, 174, 229, 288, 317, 366, 468, 497, 549

Yellow Fever Contracted at Night..... 344

